# Effects of gold and PCL- or PLLA-coated silica nanoparticles on brain endothelial cells and the blood–brain barrier

**DOI:** 10.3762/bjnano.10.95

**Published:** 2019-04-25

**Authors:** Aniela Bittner, Angélique D Ducray, Hans Rudolf Widmer, Michael H Stoffel, Meike Mevissen

**Affiliations:** 1 Division of Veterinary Pharmacology and Toxicology, Vetsuisse Faculty, University of Bern, Länggassstrasse 124, 3012 Bern, Switzerland; 2 Department of Neurosurgery, Research Unit, Inselspital, University of Bern, Freiburgstrasse 8, 3010 Bern, Switzerland; 3 Division of Veterinary Anatomy, Vetsuisse Faculty, University of Bern, Länggassstrasse 120, 3012 Bern, Switzerland

**Keywords:** blood–brain barrier, laser tissue soldering, nanomedicine, nanoparticle uptake, rBCEC4 cells

## Abstract

Nanomedicine is a constantly expanding field, facilitating and improving diagnosis and treatment of diseases. As nanomaterials are foreign objects, careful evaluation of their toxicological and functional aspects prior to medical application is imperative. In this study, we aimed to determine the effects of gold and polymer-coated silica nanoparticles used in laser tissue soldering on brain endothelial cells and the blood–brain barrier using rat brain capillary endothelial cells (rBCEC4). All types of nanoparticles were taken up time-dependently by the rBCEC4 cells, albeit to a different extent, causing a time- and concentration-dependent decrease in cell viability. Nanoparticle exposure did not change cell proliferation, differentiation, nor did it induce inflammation. rBCEC4 cells showed blood–brain barrier characteristics including tight junctions. None of the nanoparticles altered the expression of tight junctions or impaired the blood–brain barrier permeability. The findings suggest that effects of these nanoparticles on the metabolic state of cells have to be further characterized before use for medical purposes.

## Introduction

Nanotechnology is commonly used in various fields, such as agriculture and pharmaceutical industry, and has gained further importance over the past few decades [1]. This technology also offers promising possibilities for medical applications such as tumor diagnostics and therapy, as drug carriers or in biodegradable implants, e.g., in laser tissue soldering (LTS) [2].

LTS provides a promising alternative treatment method for injuries of hollow organs, e.g., vessels, offering faster procedure time, immediate watertightness, faster wound healing and reduced recovery time compared to classical microsuturing [3–5]. This technique makes use of a degradable polymer scaffold containing albumin and the chromophore indocyanine green (ICG). The latter enables the transduction of laser light into heat leading to denaturation of the albumin and, subsequently, tissue fusion [6]. As ICG is unstable in aqueous solutions and prone to fast photo-bleaching, the use of a stabilizing system, such as encapsulation in nanoparticles (NPs), increases precision and success of the procedure. Alternatively, gold NPs (Au-NPs) allow for localized and precise application of LTS [7–8].

Nanomaterials are foreign materials and, hence, might elicit adverse effects when they come in contact with bodily tissue, vessels and specialized structures such as the blood–brain barrier (BBB). To be able to safely employ LTS in nanomedicine, such unwanted effects need to be studied.

Previously, we investigated effects of silica (Si-), namely silica-ICG/poly(ε-caprolactone) (PCL) and silica-ICG/poly(ε-caprolactone-poly(L-lactide) (PLLA), and Au-NPs used in LTS on cells of the brain, namely microglial and neuron-like cells. Si-NPs were further characterized regarding their interactions with cells by using organotypic hippocampal tissue slices and primary cultures. All types of NPs were found in microglial cells and neuron-like cells in membrane-surrounded vesicles and the cytoplasm. Studies in organotypic brain slices revealed that NPs were only taken up by microglial cells but not by astrocytes or neurons [9]. NPs were taken up in a time- and concentration-dependent manner and were found in the endoplasmic reticulum and lysosomes in microglia [10]. None of the NPs investigated resulted in cytotoxicity, decreased cell viability, apoptosis, autophagy or inflammation. However, exposure to NPs led to oxidative stress via depletion of cellular glutathione and to a downregulation of neuronal differentiation markers in neurons [11]. Kamikobu et al. reported that the effect of Si-NPs on cell viability of embryonic kidney cells and primary hippocampal cultures depended on concentration, size and surface charge of the particles. Notably, neuronal cells were shown to be more sensitive to NP exposure compared to embryonic kidney cells. Si-NPs induced time- and concentration-dependent neuronal cell death by production of reactive oxygen species and reduction of glutathione levels [12]. Similarly, Si-NPs led to morphological changes, concentration-dependent membrane damage, decreased cell viability, increased apoptosis, oxidative stress and an increase in inflammatory cytokines in dopaminergic neuron-like cells. In vivo intranasal administration of these NPs corroborated these findings and showed localization of Si-NPs mainly in the striatum and hippocampus [13].

As LTS finds its application in vessels of the brain, the effects of various NPs on cells of the vasculature and the BBB need to be determined. The BBB is made of specialized endothelial cells (ECs), astrocytes and pericytes, forming a tight barrier, thus restricting access to the brain [14–15]. Disruption of this barrier allows potentially harmful molecules to enter the brain and cause or worsen diseases of the central nervous system [16] that NPs might contribute to [17].

Coated or uncoated mesoporous Si-NPs of different size and zeta potential did not elicit considerable cytotoxicity in MDCK II kidney epithelial cells or RBE4 rat brain ECs but were taken up by both cell types. However, uptake was found to be more prominent in RBE4 cells compared to MDCK II cells [18]. After exposure of rat primary cultured brain microvessel ECs (rBMECs) to Au-NPs, smaller NPs were demonstrated to be taken up to a higher extent compared to larger NPs. Overall, only the smallest Au-NPs showed an effect on cell viability. Regardless of size, none of the NPs induced inflammation or cell morphology changes [19]. This could also be shown for primary cultured porcine brain microvessel ECs (pBMECs) exposed to Au-NPs [20]. Si-NPs elicited concentration- and time-dependent cytotoxicity in HUVECs. Furthermore, Si-NPs were shown to induce oxidative stress and inflammation mediated by mitogen-activated protein kinase (MAPK) and nuclear factor kappa-light-chain-enhancer of activated B cells (NF-κB) [21] pathways that are related to cell proliferation and differentiation but also to inflammation and apoptosis via connection to the NF-κB pathway [22].

Size- and dose-dependent cytotoxicity and disruption of the BBB after exposure to SiO_2_ particles were shown in a human model and confirmed in vivo [23]. Integrity and function of the BBB of primary porcine brain microvascular ECs (PBECs) in co-culture with SH-SY5Y cells were not affected by exposure to PEGylated Au-NPs [24]. Similar results were reported by Trickler et al. using Au-NPs in both a rat and a porcine model of the BBB. Smaller Au-NPs, however, increased the BBB-permeability in the rBMEC monolayer but not the pBMEC monolayer [19].

In the context of LTS, ECs will be in direct contact with NPs being released from the degrading scaffold [25]. The same holds true for intravascular applications of NPs in general. Hence, in this study, brain ECs, rBCEC4, were used to examine possible effects of two types of Si-NPs as well as Au-NPs on cell viability, induction of inflammation and uptake and intracellular localization of NPs.

## Results

### NP effects on rBCEC4 cell viability

**Figure 1:**
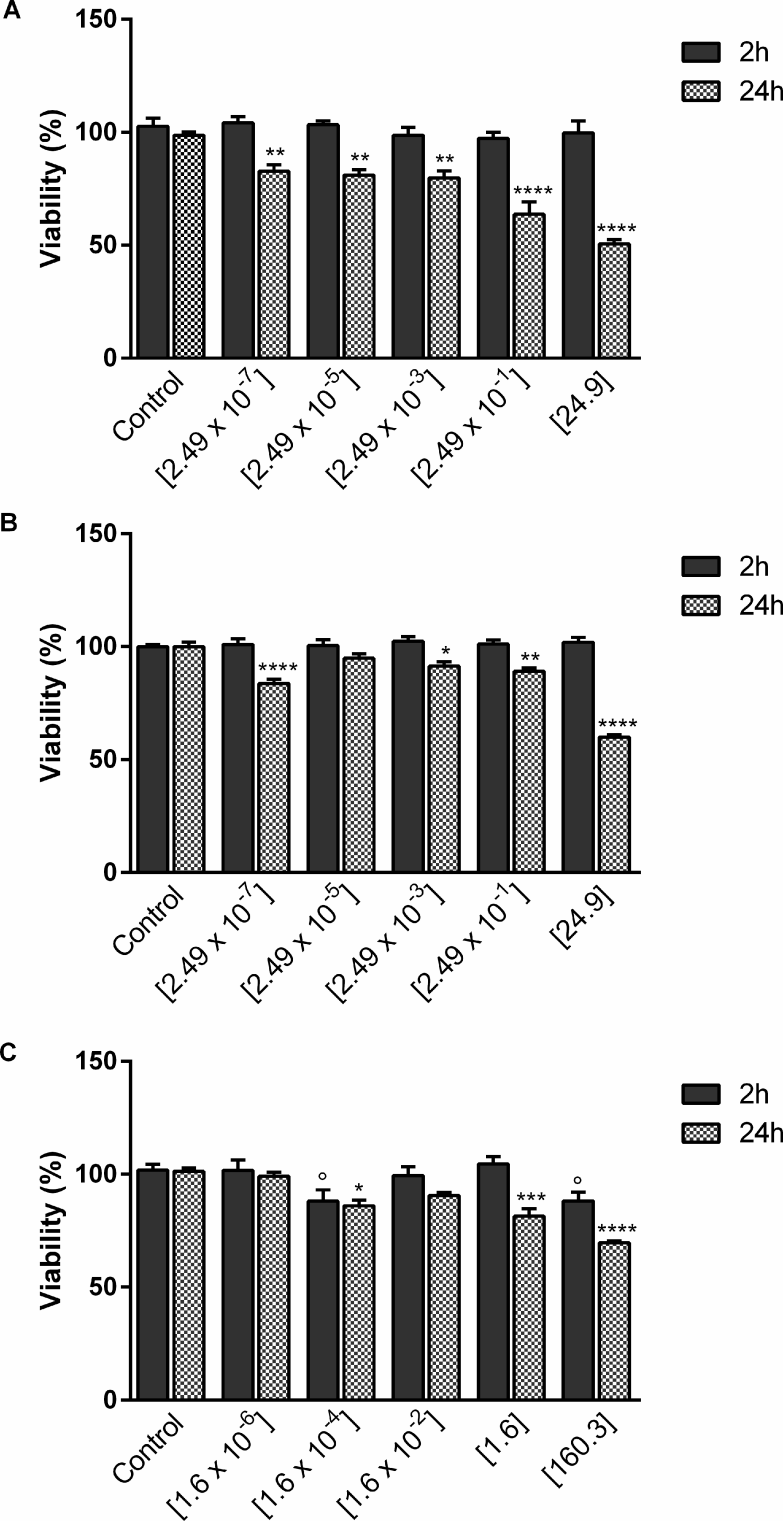
Concentration-dependent effects of PCL- (A), PLLA- (B) and Au- (C) NPs on rBCEC4 cell viability. A time- and concentration-dependent effects was detected after exposure to all three types of NPs. Concentrations of PCL- or PLLA-NPs were [2.49 × 10^−7^ µg/mL] to [24.9 µg/mL] and of Au-NPs [1.6 × 10^−6^ µg/mL] to [160.3 µg/mL] with 100-fold increases in between. Error bars represent SEM. Control: non-exposed cells. Significant differences between NP-exposed and non-exposed control are labeled with circles (°) for 2 h of NP exposure and asterisks (*) for 24 h of NP exposure, respectively (°/* = *p* ≤ 0.05; ** = *p* ≤ 0.01; *** = *p* ≤ 0.001; **** = *p* ≤ 0.0001).

rBCEC4 cells were exposed to five different NP concentrations for each of the three NP types used, namely PCL-, PLLA- and Au-NP, for 2 and 24 h (Figure 1). Regardless of the concentration, neither PCL- nor PLLA-NPs affected cell viability after 2 h of exposure even though a high percentage of cells (57% for PCL-NPs and 46% for PLLA-NPs) had taken up NPs. In contrast, Au-NP concentrations of [160.3 µg/mL] and [0.16 ng/mL] caused a significant decrease in cell viability of 10%. After 24 h of exposure, a significant effect on cell viability could be detected at the highest NP concentration resulting in a decrease of 50%, 40% and 30% for PCL-, PLLA- and Au-NPs, respectively. All NP types and concentrations resulted in a decrease in cell viability of less than 20% with the exception of exposure to [0.25 µg/mL] PCL-NP for 24 h. Overall, PCL-NPs exhibit the most pronounced effect on the viability of rBCEC4 cells as shown in Figure 1.

### NP uptake in rBCEC4 cells

**Figure 2:**
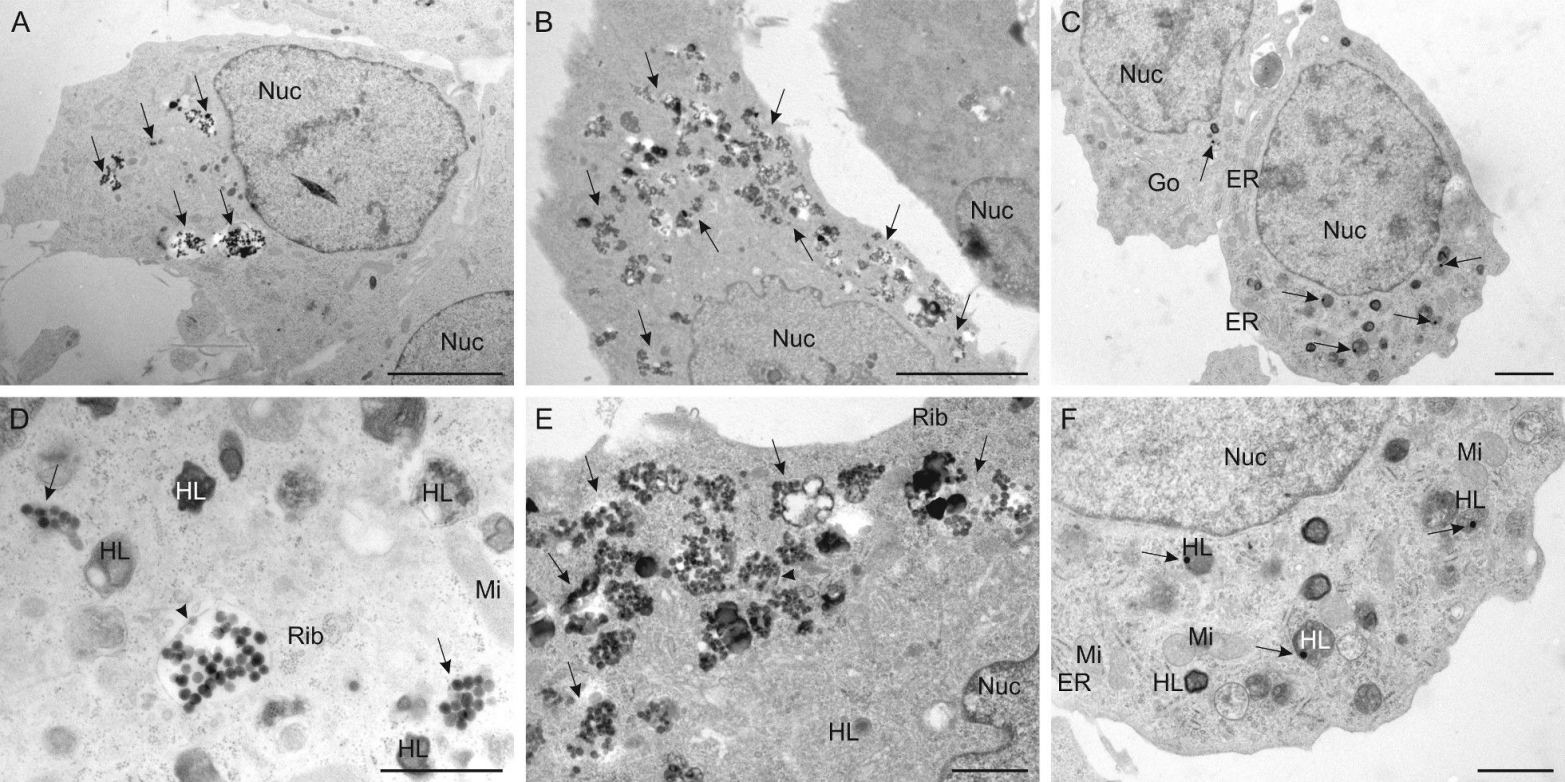
Uptake of PLLA-NPs (A, D) after 2 h of exposure and uptake of PCL-NPs (B, E) and Au-NPs (C, F) into rBCEC4 cells after 24 h of exposure; cell overview (A, B, C) and higher magnification (D, E, F); analyzed by TEM. PLLA- and PCL-NPs were found in clusters inside the cells (arrows) (A, B; scale bar: 5 µm) and were present freely in the cytoplasm (arrows) or in membrane-bound vesicles (arrow head) (D, E; scale bar: 1 µm). Single Au-NPs were taken up by the cells (arrows) (C; scale bar: 2 µm) and co-localized with heterolysosomes (arrows) (F; scale bar: 1 µm). Nuc = nucleus, HL = heterolysosomes, Rib = free ribosomes; Mi = mitochondria; Go = Golgi apparatus; ER = endoplasmic reticulum. Concentrations were [24.9 µg/mL] PLLA- or PCL-NPs and [160.3 µg/mL] Au-NPs.

Uptake of PLLA-, PCL- and Au-NPs was first investigated with TEM (Figure 2), revealing differences between these three NP types. Both PLLA- and PCL-NPs were taken up to a high extent after 2 and 24 h of exposure, respectively. They tended to form clusters and were detected freely in the cytosol or in membrane-bound vesicles (Figure 2A,D and Figure 2B,E). On the other hand, Au-NPs could not be found inside rBCEC4 cells after 2 h of exposure. Prolonging exposure to 24 h resulted in the uptake of few, single Au-NPs co-localizing with heterolysosomes as illustrated in Figure 2C,F.

**Figure 3:**
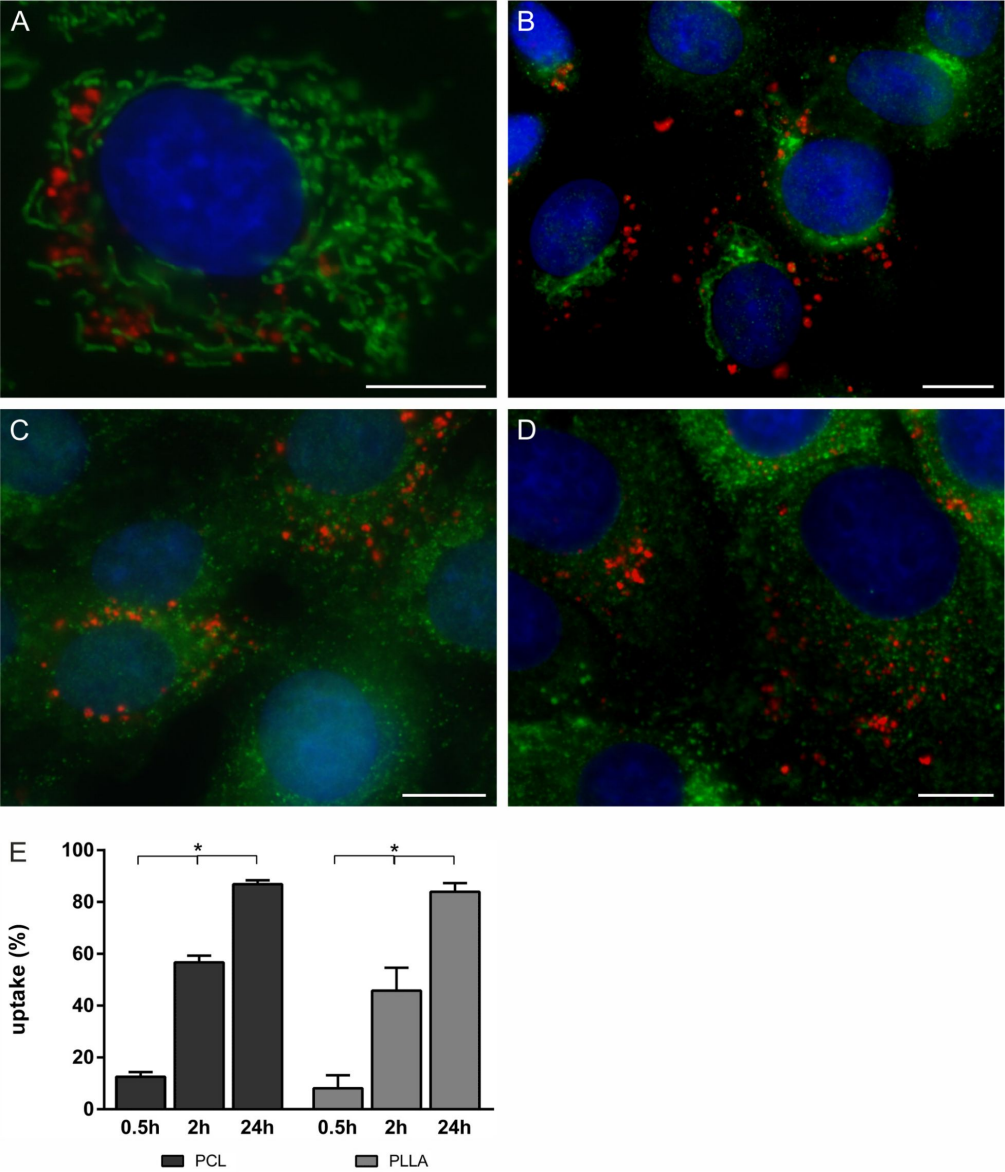
Representative microscopic images of rBCEC4 cells stained for various cell organelles (green) exposed to PCL- (A, B) or PLLA-NPs (C, D) for 24 h. No NPs (red) were found in mitochondria (ATPB-positive organelles) (A), Golgi apparatus (giantin-positive organelles) (B), lysosomes (LAMP1-positive organelles) (C) or in the ER (calreticulin-positive organelles) (D). Cell nuclei were counterstained with Hoechst (blue). Scale bars 10 µm. Uptake of PCL- and PLLA-NPs into rBCEC4 cells after 0.5, 2 or 24 h of NP exposure was quantified using high-content analysis (E). Both PCL- and PLLA-NPs showed a similar and time-dependent uptake pattern. Concentrations of PCL- or PLLA-NPs were [24.9 µg/mL]. Error bars represent SEM. Significant differences between exposure times are labeled with asterisks (*) (* = *p* ≤ 0.0001).

As both types of Si-NPs were fluorescent, NP uptake was further examined using fluorescent markers for the cytoskeleton (data not shown) and various cell organelles (Figure 3A–D). PCL- and PLLA-NPs were observed inside the cells but not in co-localization with mitochondria, the Golgi apparatus, endoplasmic reticulum or lysosomes. Both NP types were found predominantly close to the nucleus. PCL- and PLLA-NP uptake was also assessed quantitatively with high-content analysis using a fully automated inverted epifluorescence microscope (Figure 3E). As Au-NPs were only taken up to a very low extent, quantification was not carried out for these. PCL- and PLLA-NP uptake was measured after 0.5, 2 and 24 h of exposure to a concentration of [24.9 µg/mL]. A time-dependent significant increase in NP uptake was obtained in rBCEC4 cells. Both PCL- and PLLA-NPs were taken up to a very similar extent. After 24 h of NP exposure, 87% and 84% of cells had taken up PCL- and PLLA-NPs, respectively (Figure 3E).

### Signaling pathways involved in survival, proliferation and inflammation in rBCEC4 cells

Possible changes in protein expression representing inhibition or activation of several crucial proteins of different signaling pathways involved in regulatory processes including cell survival and proliferation were investigated with western blotting. The active, phosphorylated (P-) form of the proteins of interest was compared to their inactive, non-phosphorylated form.

**Figure 4:**
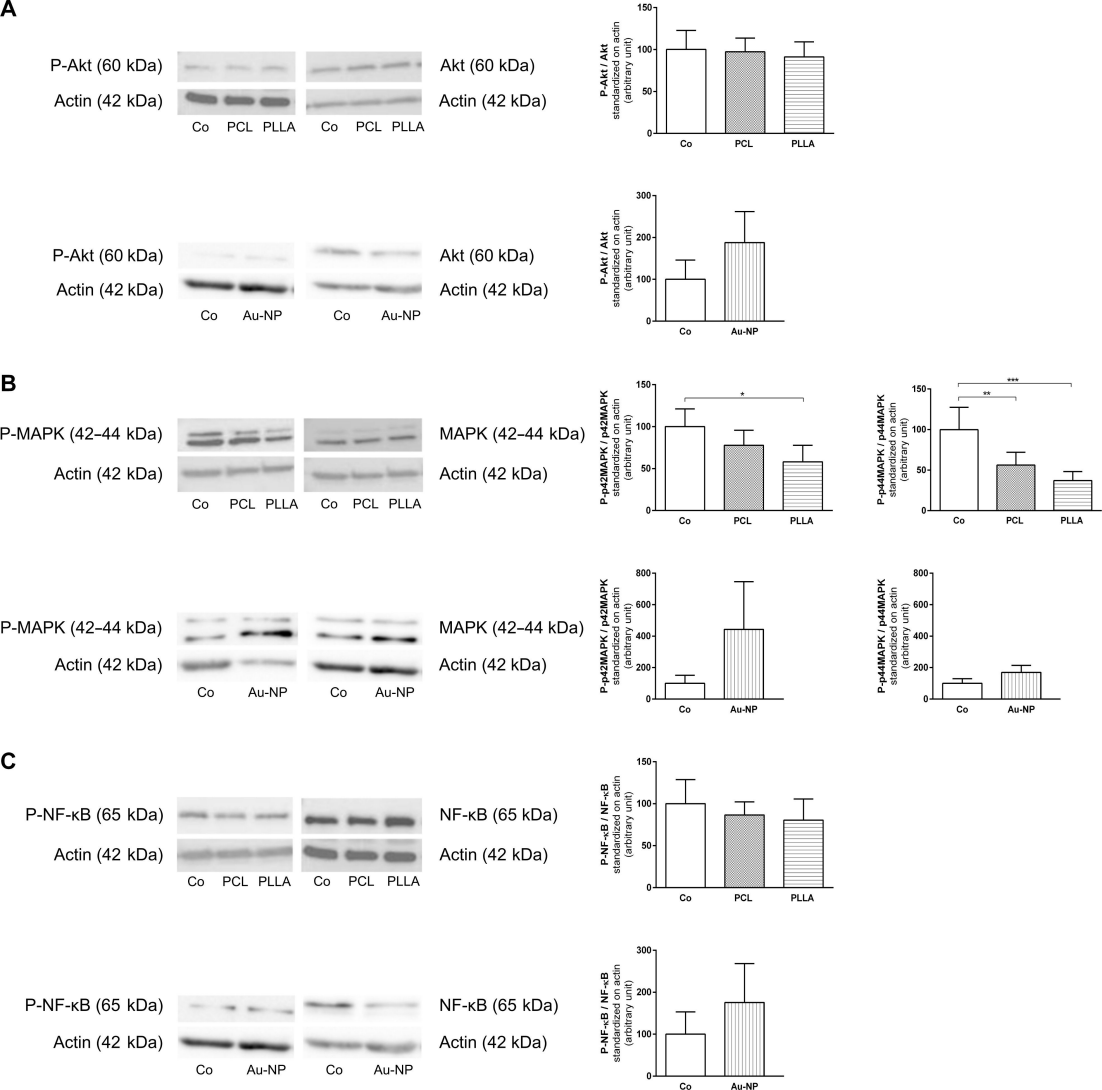
Effects of PCL-, PLLA- and Au-NP exposure on various signaling pathways. Expression of phosphorylated and unphosphorylated forms of crucial proteins of the Akt, the MAPK and the NF-κB pathway after 24 h of exposure to all three NP types was quantified and the ratios (phosphorylated/unphosphorylated) were calculated. PCL-, PLLA- and Au-NPs did not affect expression or activation of Akt or NF-κB (A, C). PCL- and PLLA-NPs led to decreased levels of P-MAPK, whereas Au-NPs caused an increase (B). Representative western blot images are depicted on the left; quantification is shown on the right. Co: control. Concentrations were [24.9 µg/mL] for PCL- and PLLA-NPs and [160.3 µg/mL] for Au-NPs. Error bars represent SEM. Significant differences between NP-exposed and non-exposed controls are labeled with asterisks (*) (* = *p* ≤ 0.05; ** = *p* ≤ 0.01; *** = *p* ≤ 0.001).

Protein kinase B (Akt) could be detected in its inactive and active form but neither exposure to Si- nor to Au-NPs caused significant changes in its expression. However, a trend to an increase in P-Akt was seen after Au-NP exposure (Figure 4A). MAPK and P-MAPK were both expressed in unexposed and exposed rBCEC4 cells. MAPK was present at similar levels under all conditions for all three NP types, whereas differences were visible in P-MAPK expression after NP exposure. PCL- and PLLA-NP exposure caused a significant decrease in phosphorylation of MAPK when compared to unexposed control cells. This decrease was more prominent in PLLA-NP-exposed cells compared to PCL-NP-exposed cells. Au-NPs on the other hand, resulted in an increase in P-MAPK that was not statistically significant, as shown in Figure 4B. Neither Si- nor Au-NPs led to differences in activation or expression of NF-κB. Both forms could be detected for this protein (Figure 4C).

### Expression of tight-junction proteins in rBCEC4 cells

**Figure 5:**
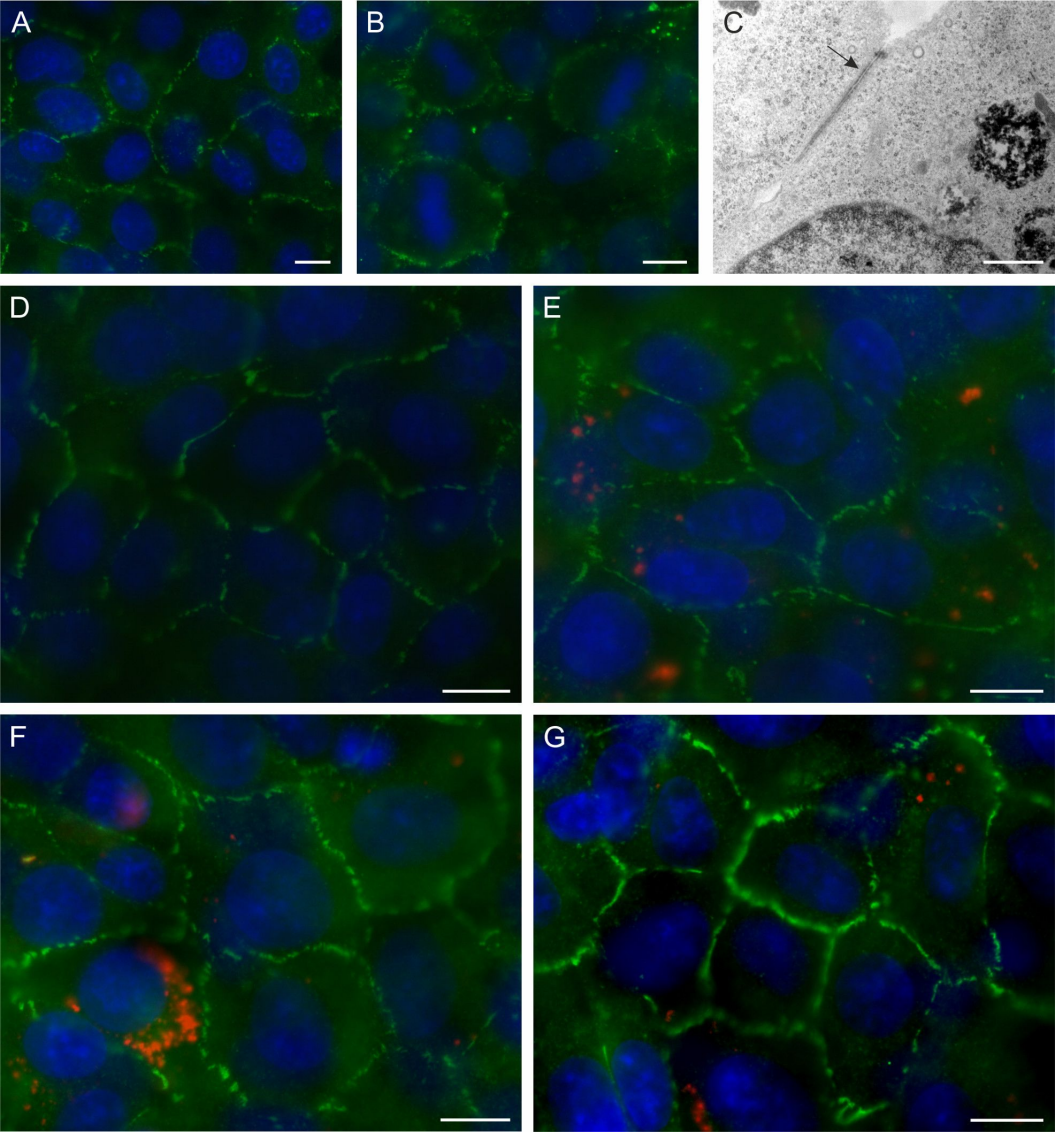
Representative microscopic images of rBCEC4 cells stained for TJs. Staining (green) shows expression of ZO-1 (A, D-G) and occludin (B) in rBCEC4 cells. TEM further confirmed existence of TJs in these cells (C). PLLA-NPs were used to investigate possible effects of NP exposure on rBCEC4 cells: control (D), PLLA-NPs were added shortly after seeding the cells (E), about 24 h before cells reached full confluence (F) and after monolayer formation (G). No difference between control and any of the three conditions of NP exposure could be detected. PLLA-NPs are shown in red. Cell nuclei were counterstained with Hoechst (blue). Concentration of PLLA-NPs was [24.9 µg/mL]. Scale bars: 10 µm (A, B, D–G). Scale bar 1 µm (C).

Immunofluorescence staining and TEM were used to demonstrate the expression of important BBB-characteristics, namely tight junction (TJ) formation, in rBCEC4 cells. Both, the TJ-associated protein zonula occludens-1 (ZO-1) and the TJ protein occludin, resulted in positive staining (Figure 5A,B). TEM pictures corroborated the formation of TJs between single rBCEC4 cells in a cell monolayer (Figure 5C).

**Figure 6:**
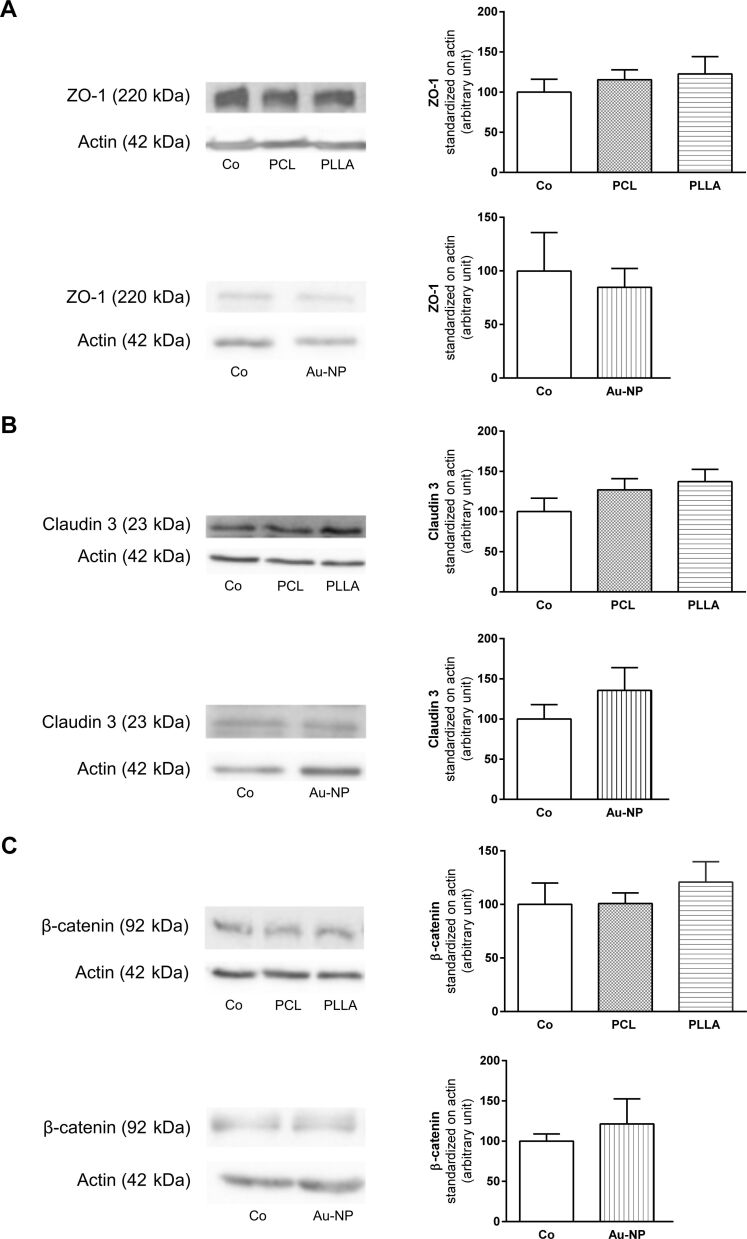
Effects of PCL-, PLLA- and Au-NPs on expression and regulation of TJs in rBCEC4 cells. Expression levels of ZO-1 (A), claudin 3 (B) and β-catenin (C) were evaluated and quantified. None of the NPs exhibited an effect on the expression levels of these proteins. Representative western blot images are depicted on the left, corresponding quantifications are shown on the right. Co: control. Concentrations were [24.9 µg/mL] for PCL- and PLLA-NPs and [160.3 µg/mL] for Au-NPs. Error bars represent SEM.

A possible effect of NP exposure on TJ formation and established TJs was investigated using immunofluorescence staining for ZO-1 (Figure 5D–G). rBCEC4 cells were exposed to PLLA-NPs at a concentration of [24.9 µg/mL] at various time points during and after monolayer and barrier formation. No differences in signal intensity or continuity of ZO-1 between control cells and any of the conditions of PLLA exposure were detected. PCL-NP exposure did not elicit changes in immunofluorescence staining of ZO-1 either (data not shown). No variations in protein levels of ZO-1 and TJ protein claudin 3 were observed after exposure to PCL-, PLLA- or Au-NPs compared to non-exposed controls (Figure 6A,B).

β-Catenin, the key player in the canonical Wnt signaling pathway, has been demonstrated to regulate and coordinate cell–cell adhesion by formation and stabilization of adherens and tight junctions [26]. The active, unphosphorylated form was expressed under all experimental conditions. The phosphorylated form could not be detected. NP exposure did not induce changes in the level of β-catenin protein expression between exposed and non-exposed cells after incubation with rBCEC4 cells for 24 hours (Figure 6C).

### Effect of NPs on blood–brain barrier permeability

**Figure 7:**
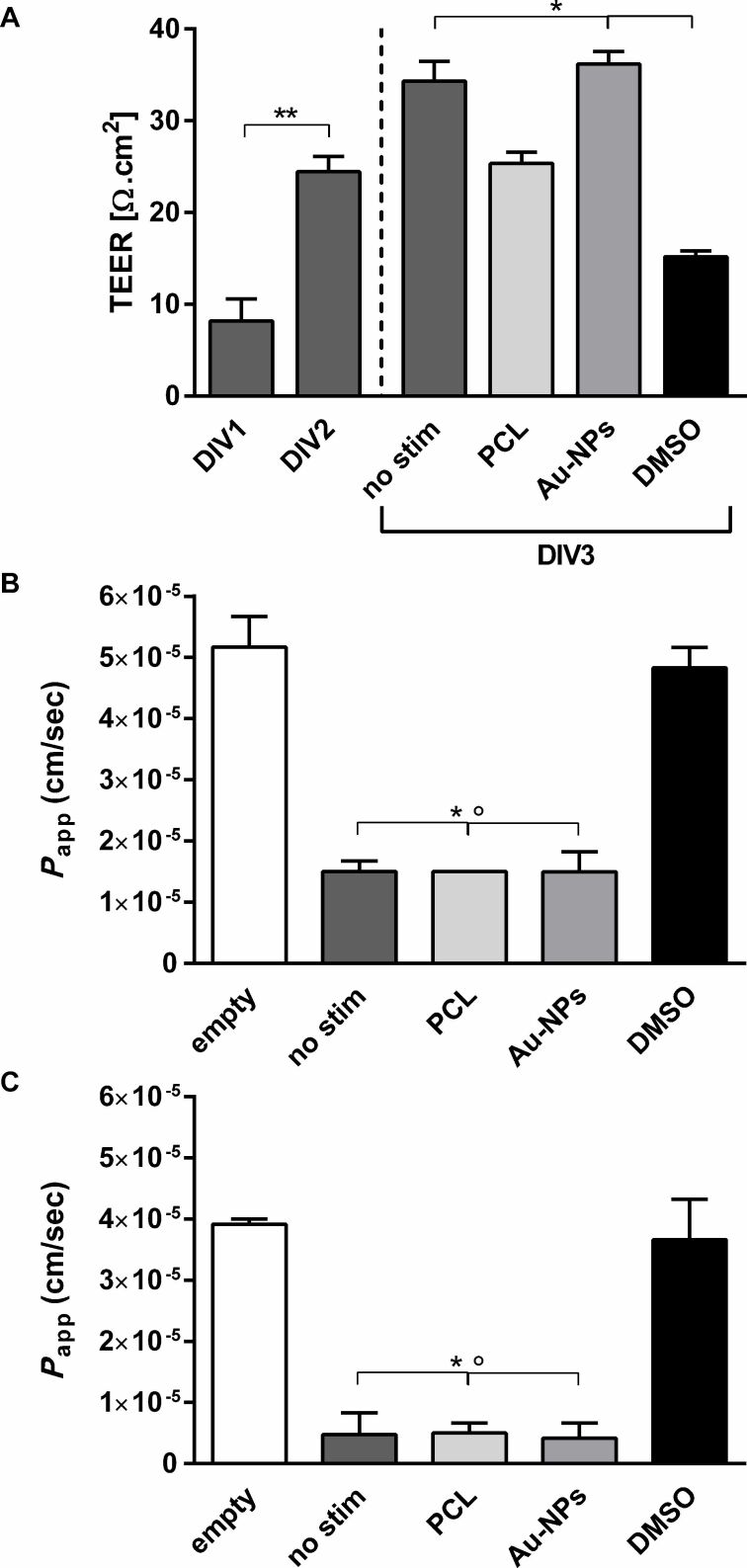
Effects of PCL- or Au-NP exposure on TEER (Ω·cm^2^) and permeability of an rBCEC4 monolayer. Permeability is indicated by *P*_app_ (cm/s). An increase in TEER was observed over the 3 days in culture (DIV: days in vitro). DMSO resulted in a significant decrease of TEER whereas NP exposure did not. The dashed line signifies the start of NP exposure and DMSO stimulation (A). None of the NPs examined led to a change in permeability, regardless of the tracer used – 4.4 kDa TRITC dextran (B) or 70 kDa FITC dextran (C). Concentrations were [24.9 µg/mL] for PCL-NPs, [160.3 µg/mL] for Au-NPs and 10% DMSO. Error bars represent SEM. Significant differences to empty filters and DMSO are labeled with asterisks (*) or circles (°), respectively (B, C). (°/* = *p* ≤ 0.01; ** = *p* ≤ 0.0001).

rBCEC4 cells were grown on filter insert membranes to allow for the investigation of NP effects on BBB permeability. The transport of two tracers across the cell monolayer and transendothelial electrical resistance (TEER) were measured. TEER measurements showed a statistically significant increase over time (Figure 7A). After NP exposure ([24.9 µg/mL] PCL-NPs or [160.3 µg/mL] Au-NPs for 24 h) and DMSO stimulation on DIV2, no changes were observed in PCL-NP-treated cell monolayers. However, as expected, DMSO stimulation resulted in a strong decrease of TEER on DIV3 as illustrated in Figure 7A. Only empty filters (no cell monolayer) and DMSO-treated rBCEC4 cell layers showed significantly increased permeability compared to filters with cells, untreated or NP-exposed. The permeability of NP-treated filters did not differ from that of untreated control filters as depicted in Figure 7B and Figure 7C. Furthermore, no difference between PCL- and Au-NP-exposed cell monolayers was detected. Overall, cell monolayers are less permeable to 70 kDa FITC dextran (Figure 7C) than to 4.4 kDa TRITC dextran (Figure 7B).

## Discussion

A concentration- and time-dependent effect of exposure to all three NP types on rBCEC4 cell viability was detected with the effect being most prominent after 24 h of PCL exposure. At similar NP concentrations, Au-NPs displayed a lower cytotoxicity for rBCEC4 cells compared to PCL- or PLLA-NPs. Contrary to this, coated or uncoated mesoporous Si-NPs of different shape, size (50 to 240 nm) and zeta potential (negative to neutral) did not elicit cytotoxicity in MDCK II kidney epithelial cells and RBE4 ECs at concentrations of up to [50 µg/mL] [18]. As the highest concentration for polymer-coated Si-NPs used in our study was half ([24.9 µg/mL]), the different effects may be due to differences in NP characteristics. PEG-*b*-PCL-NPs were highly biocompatible and did not cause significant cell viability reductions when added to hCMEC/D3 cells at concentrations of [0.01–1 mg/mL] [27]. PCL-NPs resulted in different cytotoxicity in human retinal vascular ECs, exhibiting stronger effects in the latter at concentrations of [25 µg/mL] to [200 µg/mL] with up to 50% reduction in cell viability [28], indicating that different cell types react differently to similar NPs. In line with our findings, 20 nm PEGylated Au-NPs did affect the proliferation of HUVECs up to concentrations of [100 µg/mL], a lower Au-NP concentration than the one used in our study ([160.3 µg/mL]) [24]. Unremarkable changes in cell viability were detected after exposure of rBMECs to [0.8–50 µg/mL] of various sizes of Au-NPs [19] but again, the NP concentrations used in the present study were three times higher. Besides the NP concentration, physicochemical properties, especially surface characteristics were demonstrated to be important for the interactions with cells [29–31]. Hence, variations in surface characteristics, composition and size of the NPs and the various cell types used are likely to account for differences between the studies. This is corroborated by comparisons of the effects of different types of NPs on the cell viability of different cell types [28,32].

All three NP types were taken up by rBCEC4 cells, but with variations in extent and duration demonstrated by higher and faster uptake of PCL- and PLLA-NPs than of Au-NPs. High-content analysis resulted in almost 90% of rBCEC4 cells with internalized PCL- or PLLA-NPs after 24 h of exposure. This is rather surprising and reminds of the extent of uptake in microglial cells where NPs were also found in lysosomes [10]. Previously published data showed that neurons take up NPs when they are kept in monoculture, whereas this was not the case in organotypic slice cultures, where NPs were predominantly found in microglia [9]. Therefore, the lack of other cell types in the rBCEC4 monoculture might explain the high amount of cells containing NPs. Similar to our findings, mesoporous Si-NPs were taken up by both epithelial, MDCK II, and endothelial cells, RBE4. Uptake was shown to be enhanced by copolymer coating and was found more prominent in RBE4 cells compared to MDCK II cells [18]. In contrast to our findings, Trickler et al. found a size-dependent rapid accumulation of Au-NPs in rBMECs within 30 min [19]. In agreement with the co-localization of Au-NPs with heterolysosomes found in our study, Au-NPs of the same size (80 nm) were shown to be taken up by HUVECs, localizing in endosomes and lysosomes [33]. Besides physicochemical properties, the formation of NP clusters before entry into the cell may modulate the cellular uptake [9,34].

NPs might not only cause cytotoxicity but also hinder proliferation, differentiation or lead to inflammation via activation or inhibition of various pathways including phosphatidylinositol-3-kinase/Akt (PI3K-Akt) [35], MAPK and NF-κB [22]. Hence, the expression of different key proteins after exposure to PCL-, PLLA- and Au-NPs was evaluated. P-MAPK-expression was significantly altered after exposure to PCL-NPs and PLLA-NPs but not to Au-NPs. A decrease in phosphorylation was detected in cells that had been exposed to either PCL- or PLLA-NPs with PLLA-NPs eliciting a slightly more prominent effect. Compared to this, TiO_2_-NPs caused increases in phosphorylation of Akt and all three MAPKs followed by activation of NF-κB [36]. Guo et al. demonstrated that Si-NP exposure induced inflammation in the human endothelial cell line HUVEC. The effects were mediated by the induction of phosphorylation of proteins involved in oxidative signaling and inflammation, namely two key MAPKs – JNK and p38 MAPK – and NF-κB [21]. This is in contrast to our study, in which none of the NPs investigated had a significant effect on the expression levels of Akt or NF-κB or their respective phosphorylated forms indicating that the NPs do not modulate cell proliferation and inflammation. The fact that the MTT assay measures the metabolic activity of a cell might explain the lack of alterations in markers of proliferation pathways [37–38].

For LTS in the brain, ECs representing BBB characteristics including TJs are important to allow for the investigation of the effects of NPs on BBB integrity. We could show that rBCEC4 cells express TJs and TJ-related proteins, namely occludin, claudin 3 and ZO-1. None of the NPs investigated impaired expression, integrity or functionality of TJs. In accordance with our findings, PEGylated Au-NPs did not alter TJ expression, TEER or *P*_app_ of a co-culture model consisting of PBECs and SH-SY5Y [24]. Copper and Ag-NPs on the other hand led to an increase in permeability of the PBMEC monolayer [19–20]. Liu et al. showed a size-dependent effect of SiO_2_ particles on the expression of occludin and ZO-1 and BBB permeability with particles in the nanometer range causing a decrease in TJ protein expression and an increase in permeability, whereas microparticles did not affect either [23]. Surface-modified poly(lactide-*co*-glycolide) NPs decreased TEER and increased permeability in a HBMEC–human astrocyte co-culture model [39]. Compared to a BBB model using hCMEC/D3 cells, TEER and *P*_app_ values in our model, albeit being slightly lower and higher, respectively, were comparable [40]. However, models using primary ECs resulted in higher TEER and lower overall permeability [19,23–24]. Overall, various BBB models have been established and improved over the past few decades, exhibiting varying degrees of in vivo BBB characteristics and thus variable suitability for studying certain aspects of the BBB [41–43]. This underlines the importance of carefully choosing the correct model for the intended purpose of the study.

## Conclusion

The data obtained for the assessment of effects of PCL-, PLLA- and Au-NPs used in LTS in the brain, except for reduced cell viability, do not indicate an impairment of the BBB and functional integrity in rBCEC4 cells under the given experimental conditions. The influence of NPs on the metabolic state of the cells needs to be investigated. Due to the simplistic nature of the model used, the results need to be assessed with BBB models, namely co-culture or 3D models that mimic the in vivo situation more closely.

## Experimental

### Cell culture

The immortalized rat brain capillary endothelial cell line rBCEC4 was characterized and kindly provided by Dr. Ingolf E. Blasig (Leibniz-Forschungsinstitut für Molekulare Pharmakologie, Berlin, Germany) [44]. Cells were grown on 0.1% gelatin (bovine origin; Sigma, Switzerland) in Dulbecco’s Modified Eagle Medium (DMEM; Life Technologies, UK) substituted with 10% heat-inactivated fetal bovine serum (FBS; Life Technologies, UK) and penicillin (100 units/mL) – streptomycin (100 µg/mL) (Life Technologies, UK) at 37 °C and 5% CO_2_.

### Nanoparticles

Silica-ICG/poly(ε-caprolactone) (PCL) and silica-ICG/poly(ε-caprolactone-poly(L-lactide) (PLLA) NPs were constructed and provided by Prof. Dr. Uwe Pieles, Department of Chemistry and Bioanalytics, Academy of Life Science, Switzerland. Both types of NPs consisted of a silica-core doped with rhodamine to enable visualization via fluorescence microscopy, followed by a layer of PCL/ICG and a surface coating with either PCL or PLLA. Characterization of these particle types showed a size of 90 nm for PCL-NPs and 95 nm for PLLA-NPs. The zeta potential was −25.4 mV and −15.9 mV for PCL- and PLLA-NPs, respectively [45]. To achieve homogenous distribution of Si-NPs in solution, both PCL- and PLLA-stock solutions were sonicated prior to incubation with cells. Sonication was carried out for 4 min at 30% amplitude on ice followed by a 5 min pause, and repeated three times. PCL- and PLLA-NP-stock solutions were then diluted 1:10 in cell culture medium, resulting in concentrations of 2.9 × 10^10^ PCL-NPs in 1 mL culture medium and 2.6 × 10^10^ PLLA-NPs in 1 mL culture medium. These concentrations correspond to [24.9 µg/mL].

Au-NPs exhibiting size and surface characteristics similar to those of the Si-NPs used were purchased from Nanopartz (Nanopartz Inc., USA). They were 80 nm in diameter, with a zeta potential of −35 mV. Au-NPs were sonicated for 5 min in a sonication bath and vortexed for 2 min prior to dilution in cell culture medium. rBCEC4 cells were exposed to a final concentration of 3.55 × 10^10^ Au-NPs per 1 mL culture medium ([160.3 µg/mL]).

We chose to use the highest concentrations of PCL- ([24.9 µg/mL]), PLLA- ([24.9 µg/mL]) and Au-NPs ([160.3 µg/mL]) for all experiments except cell viability to make sure possible adverse effects would be detected.

### Cell viability

The effect of PCL-, PLLA- and Au-NP exposure on the viability of rBCEC4 cells was examined using the methylthiazolyldiphenyl-tetrazolium bromide (MTT) (Sigma, Switzerland) assay. Cells were seeded at 10,000 cells (96-well plate), left to adhere and subsequently exposed to PCL-, PLLA- and Au-NPs for 2 h or 24 h. Non-exposed cells were used as control. At the end of NP exposure, exposed and non-exposed cells were incubated with MTT dissolved in phosphate-buffered saline (PBS) (Life Technologies, UK) for 4 h at 37 °C and 5% CO_2_ (final concentration: [0.5 mg/mL]). The cell culture medium was removed and the remaining MTT-formazan was dissolved in DMSO (Sigma, Switzerland). Absorbance was measured at 540 nm using a plate reader (Synergy HT, BioTek, Switzerland).

### Nanoparticle uptake

PCL-, PLLA- and Au-NP uptake was investigated using transmission electron microscopy (TEM). PCL- and PLLA-NP uptake was further examined by 3D-structured illumination microscopy and quantified with high-content analysis. Briefly, rBCEC4 cells were seeded at 36,000 cells (96-well plate) or 180,000 cells (24 well plate), left to adhere, then exposed to either PCL-, PLLA- or Au-NPs for either 30 min, 2 h or 24 h at 37 °C and 5% CO_2_. Subsequently, the uptake of PCL- and PLLA-NPs was assessed using a Zeiss Axio Imager Z1 plus Apotome 1 (Carl Zeiss Vision Swiss AG, Feldbach, Switzerland) and evaluated quantitatively by high-content analysis with the IN Cell Analyzer 2000 (GE Healthcare Life Sciences, USA) [10,46].

### Immunofluorescence staining and transmission electron microscopy

After PCL- or PLLA-NP exposure, cells grown in 0.1% gelatin-coated 96-well plates or on coverslips were fixed with cold 4% paraformaldehyde for 20 min at room temperature. Following two washing steps with Dulbecco’s phosphate-buffered saline (DPBS) (Life Technologies, UK), cells were blocked with 10% horse serum in 0.4% Triton-PBS for 1–2 h at room temperature. Subsequently, cells were incubated with primary antibodies overnight at 4 °C (Table 1). After washing four times with PBS, except for Acti-stain 488 phalloidin, the corresponding secondary antibodies were applied and left for 2 h before cells were washed again four times with PBS. Thereafter, coverslips were mounted on glass slides using Glycergel Mounting Medium (Dako, Denmark/USA). The samples were examined and images were obtained using a Zeiss Axio Imager Z1 coupled with an Apotome 1 (Carl Zeiss Vision Swiss AG, Feldbach, Switzerland).

**Table 1:** Antibodies.

antibody	host	company	method	dilution

primary				

giantin	rabbit	Abcam	IF^c^	1:250
LAMP1	rabbit	Abcam	IF	1:50
calreticulin	rabbit	Abcam	IF	1:500
ATPB	mouse	Abcam	IF	1:100
ZO-1/TJP1	mouse	ThermoFisher	IF/WB^d^	1:100/1:500
occludin	rabbit	Invitrogen	IF/WB	1:100/1:500
Akt	rabbit	Cell Signaling	WB	1:1000
phospho-Akt	rabbit	Cell Signaling	WB	1:1000
pMAPK	mouse	Cell Signaling	WB	1:1000–1:2000
phospho-pMAPK	mouse	Cell Signaling	WB	1:1000–1:2000
NF-κB	rabbit	Cell Signaling	WB	1:1000
phospho-NF-κB	rabbit	Cell Signaling	WB	1:1000
β-catenin	rabbit	Cell Signaling	WB	1:500
phospho-β-catenin	rabbit	Cell Signaling	WB	1:500
claudin 3	rabbit	Abcam	WB	1:500
β-actin	mouse	Sigma Aldrich	WB	1:10,000–1:20,000

secondary				

anti-rabbit IgG AF 488^a^	donkey	Invitrogen	IF	1:200–1:500
anti-mouse IgG AF 488	donkey	Invitrogen	IF	1:200–1:500
anti-rabbit IgG HRP^b^	donkey	Novex	WB	1:5000–1:10,000
anti-mouse IgG HRP	donkey	Novex	WB	1:5000–1:50,000

other				

Hoechst	—	Life Technologies	IF	1:10,000
Acti-Stain Phalloidin 488	—	Cytoskeleton	IF	1:50

^a^AF = Alexa Fluor, ^b^HRP = horseradish peroxidase, ^c^IF = immunofluorescence, ^d^WB = western blotting.

To further study uptake of PCL-, PLLA- and Au-NPs, rBCEC4 cells were seeded at 150,000 cells (24- well plate), coated with 0.1% gelatin and were incubated in cell culture medium at 37 °C and 5% CO_2_ until full confluence was reached. Cells were then exposed to PLLA-NPs for 2 h, to PCL-NPs for 24 h and Au-NPs for 2 and 24 h. Subsequently, TEM was performed as previously described [11].

### Western blotting or protein analysis

Cells were grown in T75-flasks, exposed to PCL-, PLLA- and Au-NPs for 24 h and kept until they had grown to full confluence before extracting protein as described previously [9]. Equal amounts of protein from each sample were loaded and separated on 10% to 16% SDS-PAGE gels and subsequently transferred onto PVDF membranes. The membranes were blocked for 2 h in blocking solution (5% milk in PBS and 0.2% Tween), then incubated with the respective primary antibody overnight at 4 °C on a shaker (Table 1). Following, the membranes were washed four times, incubated with the corresponding secondary antibody for 2 h at room temperature, and washed again. Quantification was carried out with ImageJ by measuring the intensity of the bands, given in arbitrary units, and subsequent standardization on actin.

### rBCEC4 monolayer permeability

rBCEC4 cells (90,000) were seeded per gelatin-coated Millicell^®^ culture plate insert with 3 µm pore size (Merck Millipore, Germany) on days in vitro 0 (DIV0) and left to grow until monolayer formation on DIV3. Except for control filters, cells were exposed for 24 h to either PCL- or Au-NPs or stimulated with 10% DMSO (positive control) on DIV2. Transendothelial electrical resistance (TEER) was measured on DIV1 to DIV3 using the Millicell ERS-2 volt ohm meter (Merck Millipore, Germany). TEER (Ω·cm^2^) of the cell monolayer was calculated according to Equation 1 [47]:

[1] $$\text{TEER}\left(\Omega \cdot {\text{cm}}^{2}\right)=\left(R_{\text{Total}}-R_{\text{Blank}}\right)\left(\Omega \right)\times A_{\text{Membrane}}\left({\text{cm}}^{2}\right)$$

*R*_Total_ is the resistance across the rBCEC4 cell layer on the coated filter membrane, *R*_Blank_ is the resistance across an empty filter membrane (only coating, no cells) and *A*_Membrane_ is the surface area of the filter membrane.

The permeability assay on DIV3 using 4.4 kDa tetramethylrhodamine isothiocyanate (TRITC) dextran and 70 kDa fluorescein isothiocyanate (FITC) dextran ([0.5 mg/mL]; Sigma, Switzerland) was carried out as described previously [48]. Briefly, transport buffer (TB) was prepared with HEPES-buffered Hank’s Balanced Salt Solution (HBSS) (Sigma, Switzerland). Inserts were transferred to 24-well plates containing TB and filled with dextran solution. After 60 min the fluorescence of samples from the donor and receiver solution at different time points was detected at 492 nm (excitation), 518 nm (emission) and 550 nm, 580 nm for 70 kDa FITC-dextran and 4.4 kDa TRITC-dextran, respectively.

The apparent permeability coefficient (*P*_app_; cm/s) was calculated according to Equation 2:

[2] $$P_{\text{app}}=\left(k\times V_{\text{R}}\right)/\left(A\times 60\right)$$

*k* is the transport rate defined as the slope obtained by using linear regression on cumulative fraction absorbed (FAcum) plotted versus time, *V*_R_ is the volume in the receiver chamber and *A* the surface area of the filter membrane. FAcum was calculated from Equation 3:

[3] $$\text{FAcum}=C_{\text{R}i}/C_{\text{D}i}$$

*C*_R_*_i_* is the concentration in the receiver chamber at the end of interval *i* and *C*_D_*_i_* the concentration in the donor chamber at the beginning of interval *i*.

### Statistical analysis

Statistical analysis was carried out with GraphPad Prism (GraphPad Software Inc., La Jolla, USA). One-way ANOVA was conducted, followed by Tukey’s multiple comparisons test for all experiments performed. *P*-values ≤ 0.05 were considered statistically significant. All experiments were done in triplicates and repeated two to three times. Results are given as mean, error bars indicate standard error of the mean (SEM).
